# Serial Point-of-care Echocardiography Performed by an Emergency Physician to Guide Thrombolytic Management of Massive Pulmonary Embolism

**DOI:** 10.7759/cureus.7771

**Published:** 2020-04-21

**Authors:** Annie Au, Patrick Hsu, Matthew McClure, Gabriel Cabrera, Eric J Kalivoda

**Affiliations:** 1 Emergency Medicine, Hospital Corporation of America West Florida GME Consortium/Brandon Regional Hospital, University of South Florida Morsani College of Medicine, Brandon, USA

**Keywords:** pulmonary embolism, point-of-care ultrasound, echocardiography, emergency medicine, triscuspid annular plane systolic excursion, systemic thrombolysis

## Abstract

Massive pulmonary embolism (PE) is a life-threatening condition with a high mortality burden. The rapid diagnosis of PE can be supported with focused cardiac ultrasound (FOCUS) by identifying signs of right ventricular dysfunction (RVD). This case report describes a patient with hemodynamically unstable massive PE who received systemic thrombolytic therapy. Emergency physicians performed serial FOCUS examinations to assess the resolution of RVD in guidance of clinical management.

## Introduction

The clinical management of a patient with massive pulmonary embolism (PE) is time sensitive and requires prompt diagnosis in the emergency department (ED) [1,2]. Despite aggressive resuscitation efforts, massive PE carries significant mortality approaching 50% [3,4]. Point-of-care focused cardiac ultrasound (FOCUS) is an invaluable bedside tool to identify signs of right ventricular dysfunction (RVD) that collectively are highly specific for the diagnosis of PE [5,6]. Multiple studies have demonstrated that emergency physicians (EPs) can use FOCUS to accurately detect RVD, including right ventricular dilatation (right ventricular to left ventricular ratio > 1:1), interventricular septal flattening, McConnell’s sign (right ventricular mid-basal and mid-apical wall hypokinesis/akinesis with apical hyperkinesis), and tricuspid annular plane systolic excursion (TAPSE) [7-12]. There are no studies that have thoroughly investigated implementing serial FOCUS examinations to determine the acute clinical response after systemic thrombolytic therapy for massive PE. Two previous reports have described utilizing FOCUS to assess the changes in right ventricular dilatation and interventricular septal flattening after thrombolytic therapy in cases of submassive and massive PE [13,14]. This case describes EP-performed serial FOCUS in evaluating the dynamic resolution of TAPSE following successful thrombolytic therapy in a patient with a massive PE.

## Case presentation

A 58-year-old male with a past medical history of diabetes, hypertension, hyperlipidemia, and tobacco use was brought in by ambulance from home to the ED with a chief complaint of syncope. His syncopal event occurred within one hour prior to arrival while he was having a bowel movement with preceding lightheadedness and dyspnea. On arrival, he endorsed complaints of dyspnea, posterior left knee pain, and recent immobility following left hip surgery approximately two weeks ago. He denied chest pain, palpitations, hemoptysis, lower extremity swelling, history of malignancy, or recent trauma. He also denied abdominal pain, nausea, vomiting, flank pain, back pain, fevers, chills, or myalgias.

On arrival to the resuscitation bay, the patient was afebrile, blood pressure of 75/26 mmHg, heart rate 119 beats per minute, respiratory rate 26 breaths per minute, and oxygen saturation of 92% on 6 L of oxygen by nasal cannula. On physical exam, he was in moderate distress and diaphoretic, airway was intact, heart sounds were normal with a regular rate and rhythm, no jugular venous distention, lungs were clear to auscultation bilaterally without wheezing or rales, capillary refill was delayed, 2+ bilateral symmetric pitting edema was appreciated with skin changes consistent with venous stasis in his lower extremities, and a left lower extremity immobilizer was in place.

**Figure 1:**
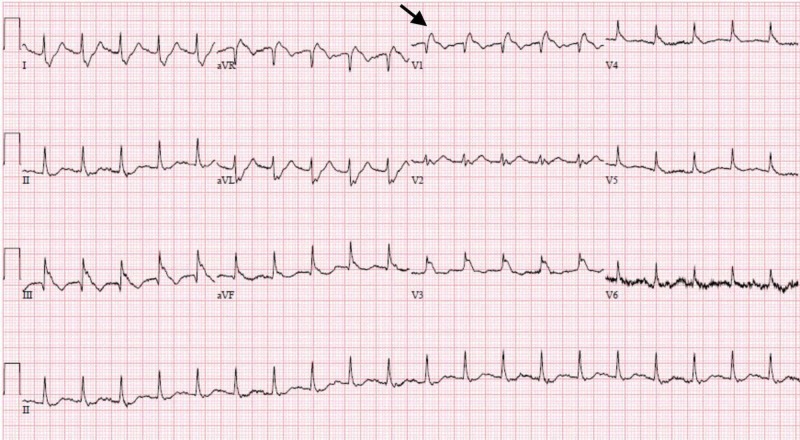
Initial electrocardiogram pre-thrombolysis demonstrating sinus tachycardia, right heart strain pattern, and right bundle branch block (black arrow).

**Figure 2:**
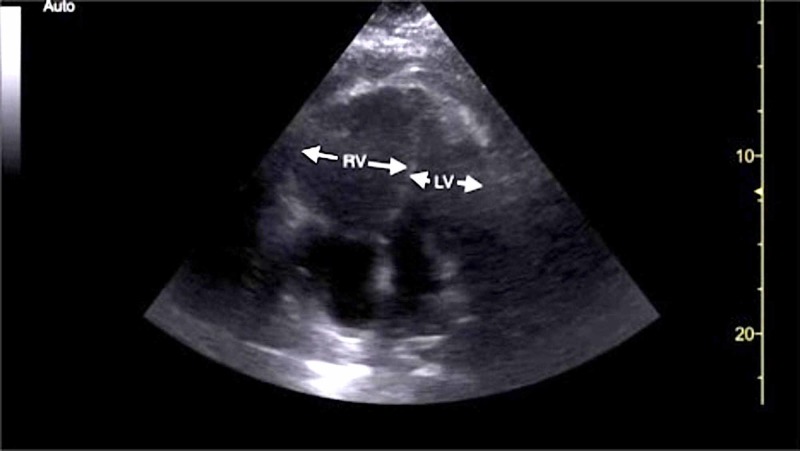
Point-of-care echocardiography pre-thrombolysis. Apical four-chamber view demonstrating RV dilatation (white arrows). LV, left ventricle; RV, right ventricle.

**Figure 3:**
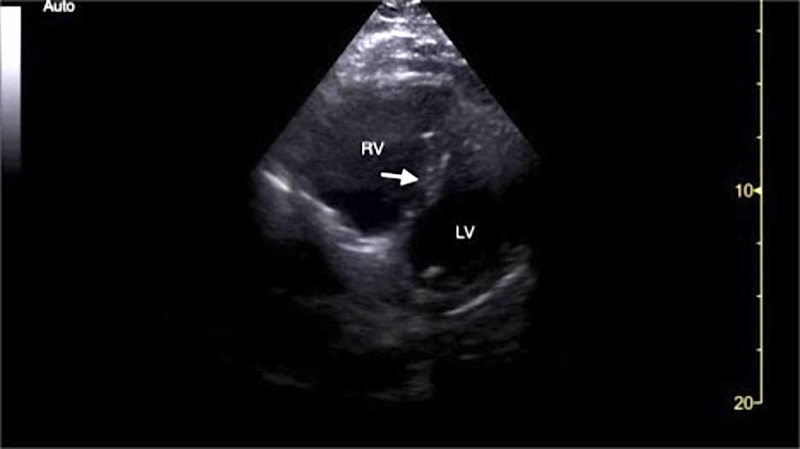
Point-of-care echocardiography pre-thrombolysis. Parasternal short-axis view demonstrating RV dilatation and interventricular septal flattening (white arrow). LV, left ventricle; RV, right ventricle.

**Figure 4:**
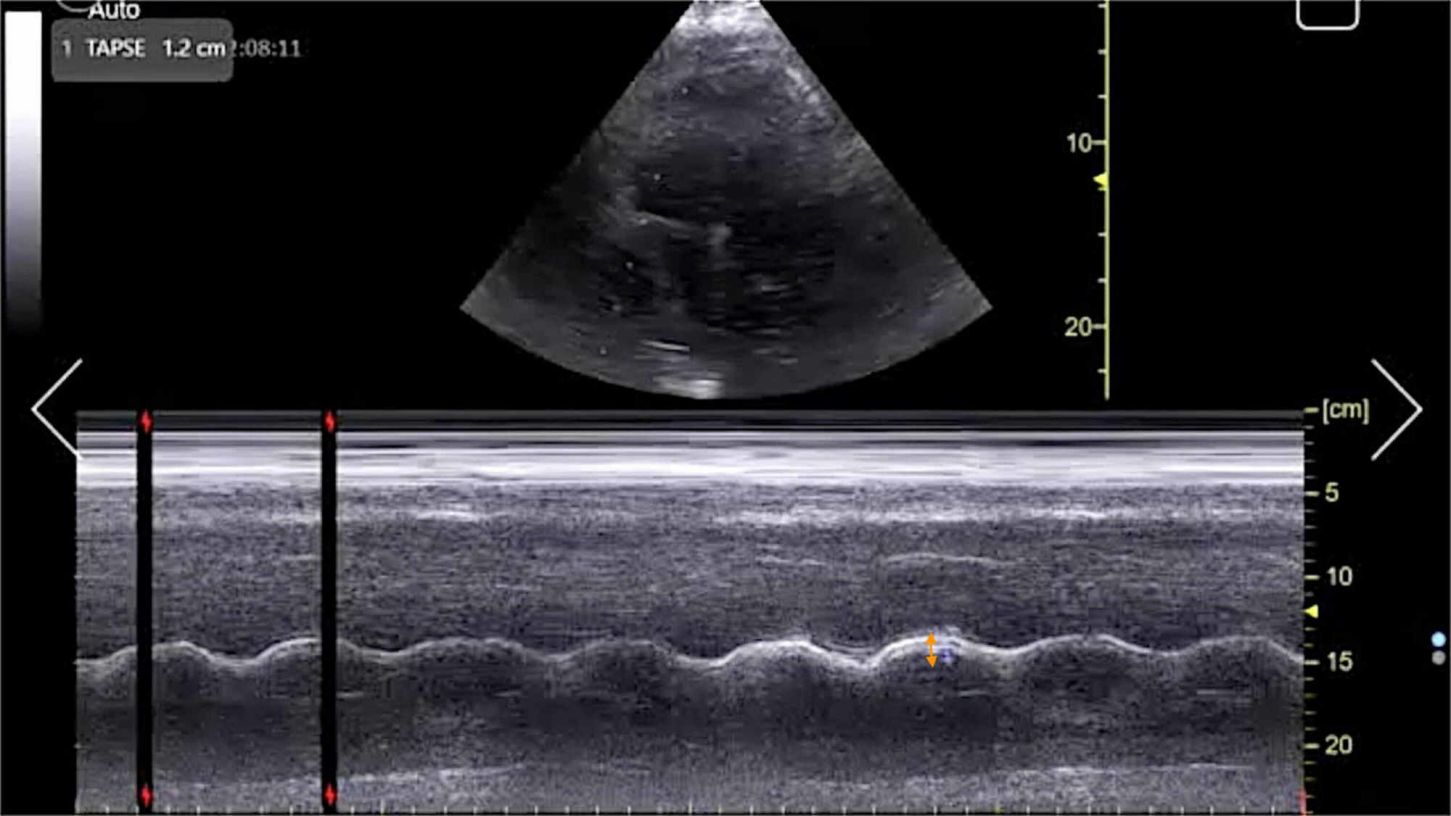
Point-of-care echocardiography pre-thrombolysis. Apical four-chamber view with M-mode tracing demonstrating TAPSE (orange arrow). TAPSE, tricuspid annular plane systolic excursion.

**Figure 5:**
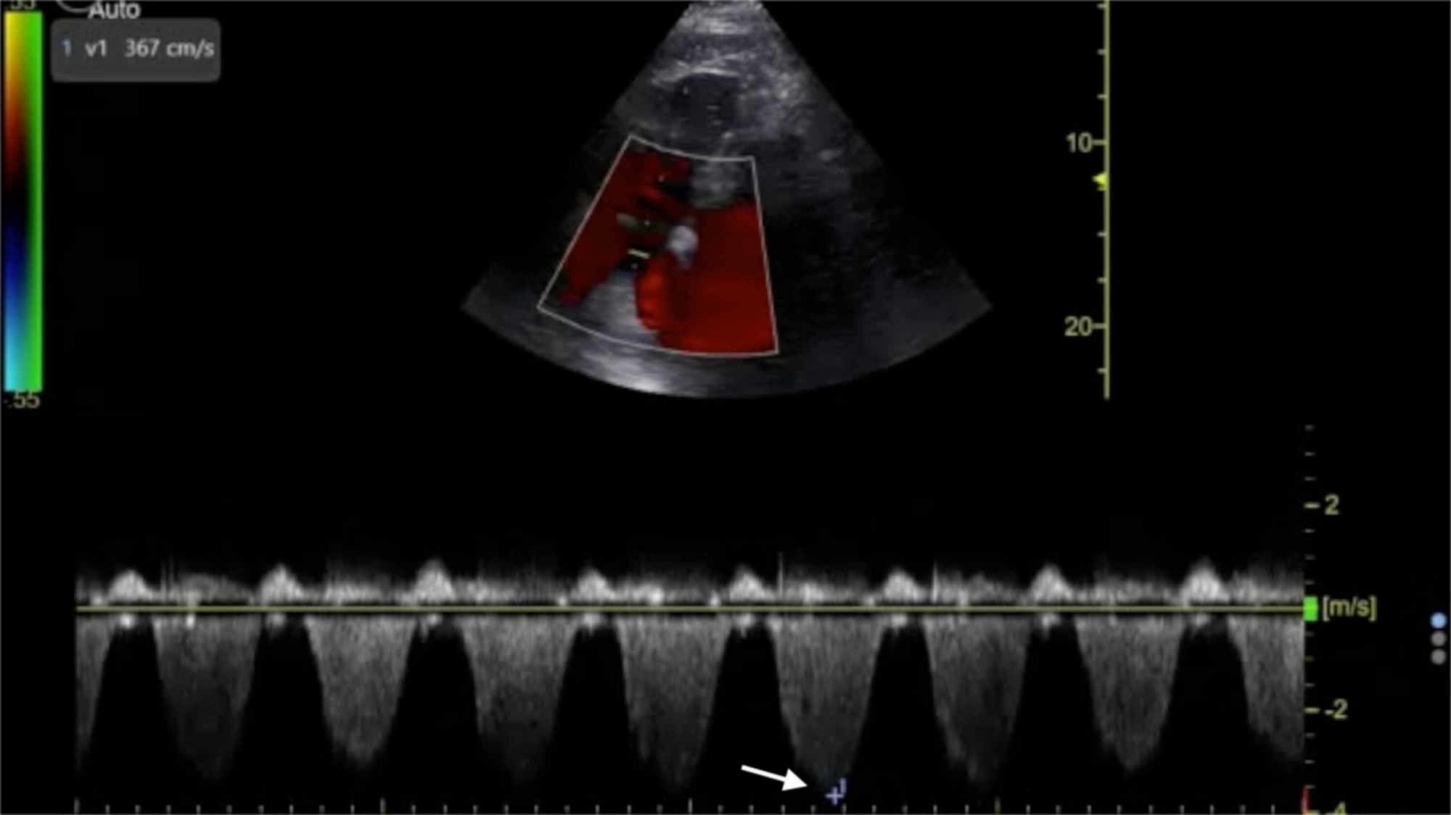
Point-of-care echocardiography pre-thrombolysis. Apical four-chamber view with Doppler tracing demonstrating maximal tricuspid regurgitation velocity (white arrow).

Initial electrocardiogram (EKG) demonstrated sinus tachycardia, right heart strain pattern, and right bundle branch block (Figure 1). Portable chest radiography revealed mild cardiomegaly without pulmonary edema or other acute abnormalities. His clinical presentation was highly concerning for acute PE and a bedside FOCUS was performed by an ultrasound fellowship-trained attending EP, which demonstrated evidence of RVD (Video 1), including right ventricular dilatation (Figure 2), interventricular septal flattening (Figure 3), diminished TAPSE (Figure 4), and increased peak tricuspid regurgitant velocity (TRV) (Figure 5). Point-of-care bedside compression ultrasound for deep vein thrombosis (DVT) was also performed which did not demonstrate any evidence of DVT in the bilateral proximal lower extremities from common femoral vein to popliteal vein. Laboratory analysis revealed pro-brain natriuretic peptide 137 pg/mL (0-125 pg/mL), troponin I 0.016 ng/mL (0-0.045 ng/mL), and a lactic acid 4.9 mmol/L. 

**Video 1:** Point-of-care echocardiography of massive pulmonary embolism performed prior to systemic thrombolysis. Apical four-chamber view demonstrating right ventricular dilatation and McConnell’s sign.

**Figure 6:**
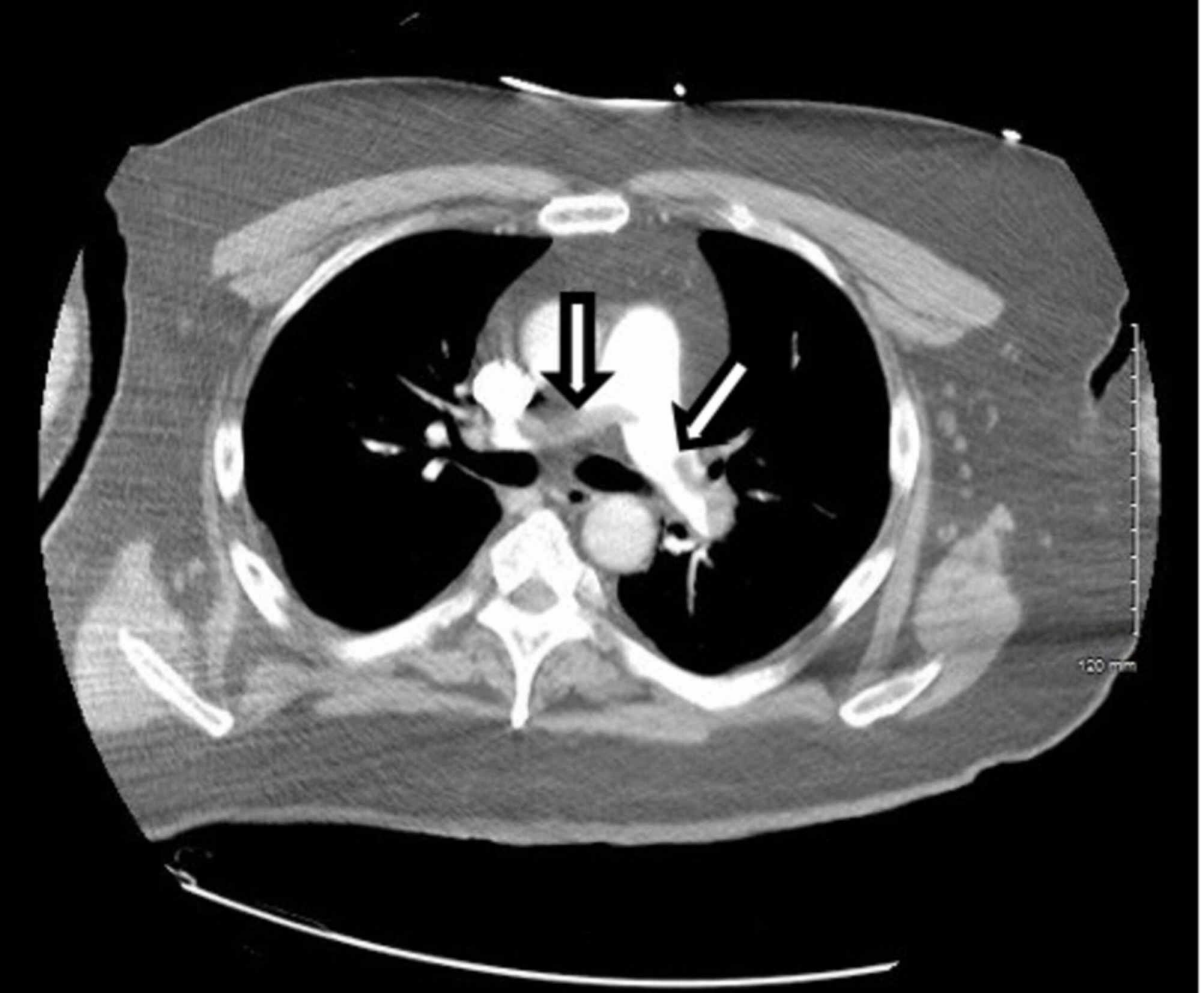
CTPA demonstrating a saddle embolus in the main pulmonary artery and emboli involving the right and left main pulmonary arteries (arrows). CTPA, computed tomography pulmonary angiogram.

**Figure 7:**
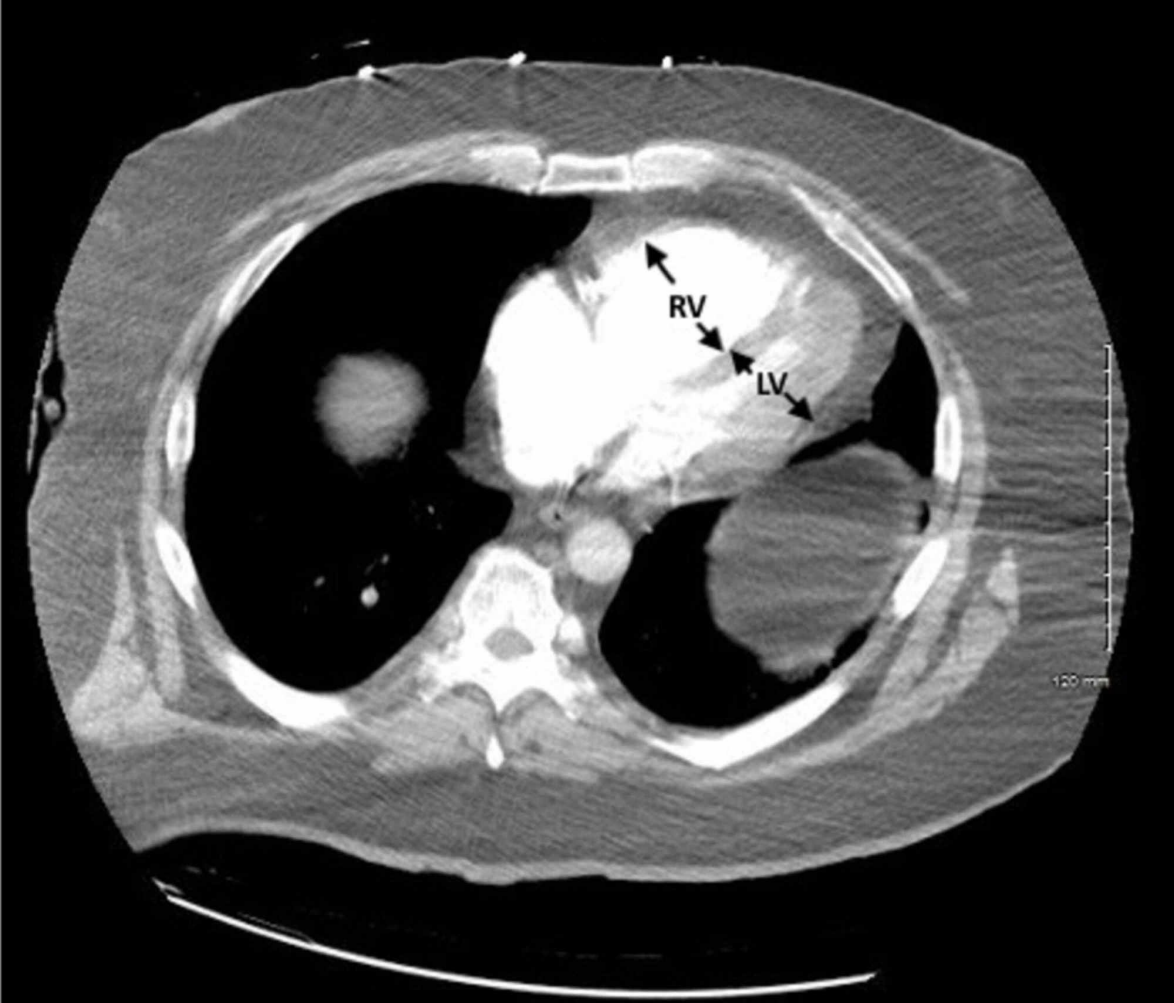
CTPA demonstrating evidence of right heart strain with RV dilatation (arrows). CTPA, computed tomography pulmonary angiogram; LV, left ventricle; RV, right ventricle.

The patient required stabilization and aggressive resuscitation with intravenous fluids and a continuous norepinephrine infusion. Based upon the FOCUS findings, a heparin drip was initiated prior to obtaining computed tomography pulmonary angiography (CTPA) which ultimately demonstrated a saddle embolus in the main pulmonary artery and emboli involving the right and left main pulmonary arteries (Figures 6, 7). 

**Figure 8:**
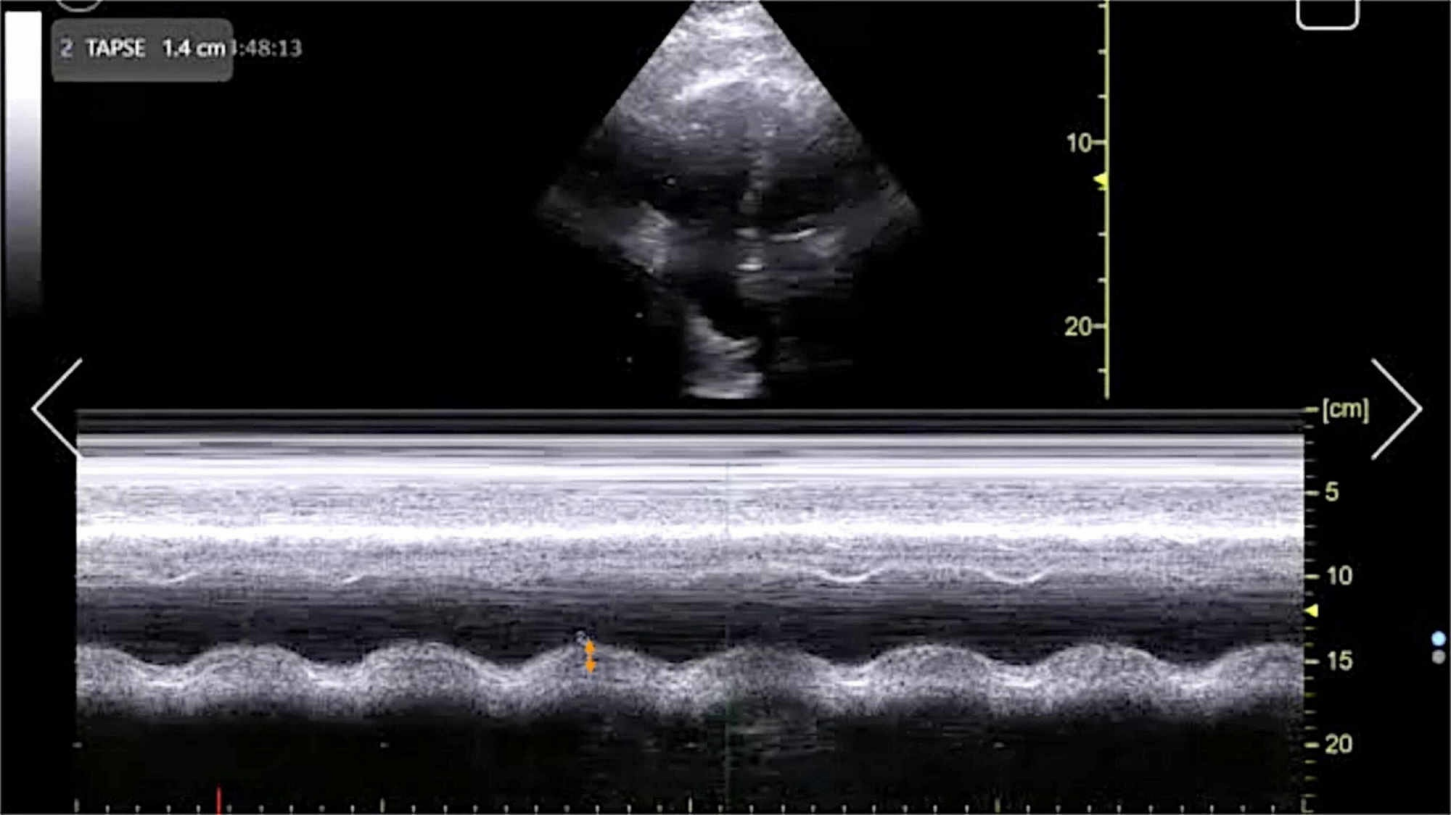
Point-of-care echocardiography two hours post-thrombolysis. Apical four-chamber view with M-mode tracing demonstrating TAPSE (orange arrow). TAPSE, tricuspid annular plane systolic excursion.

**Figure 9:**
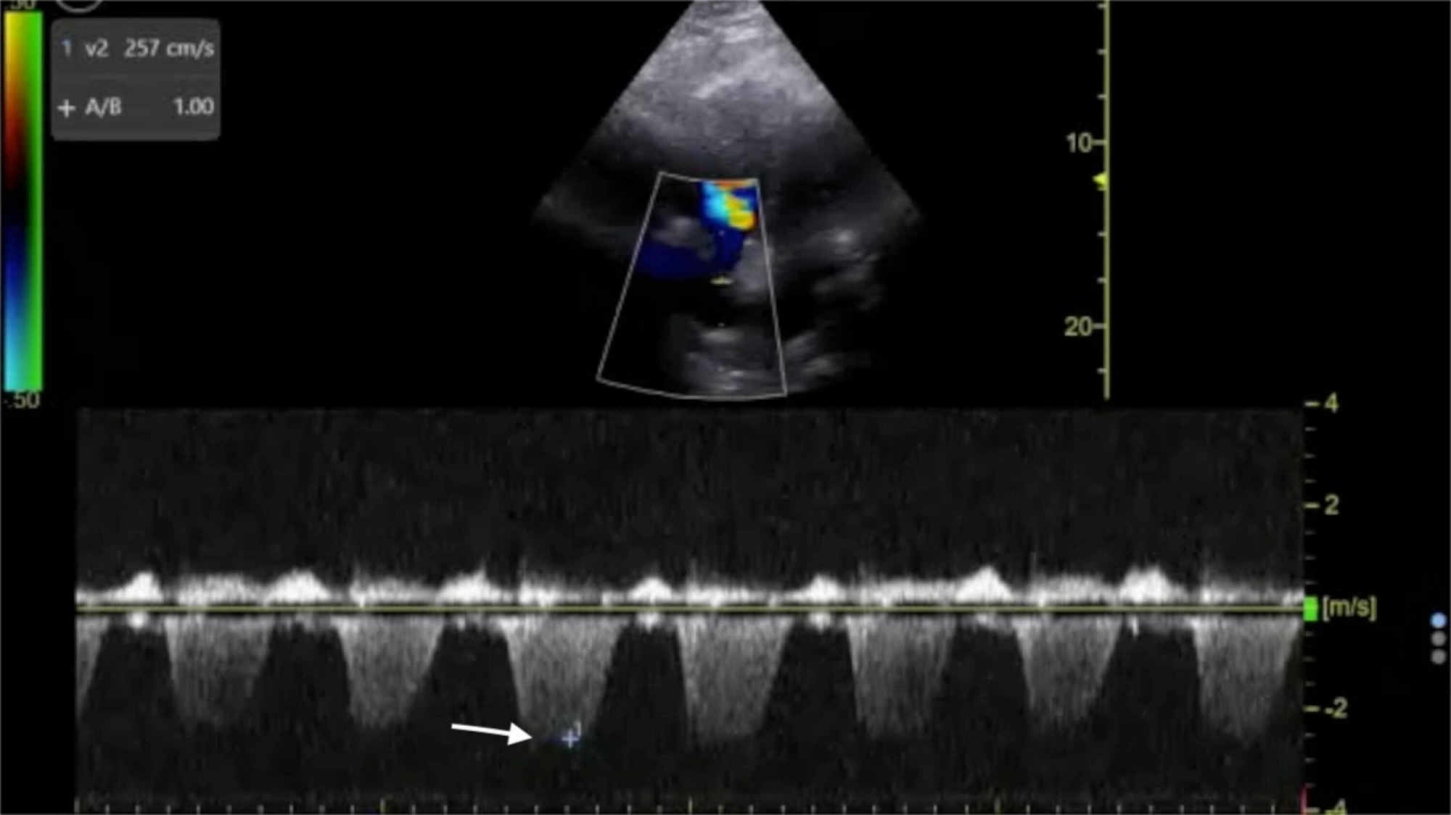
Point-of-care echocardiography two hours post-thrombolysis. Apical four-chamber view with Doppler tracing demonstrating maximal tricuspid regurgitation velocity (white arrow).

The patient and his wife at bedside chose to proceed with systemic thrombolysis after a lengthy discussion of the potential risks and benefits. A two-hour infusion of 100 mg alteplase (tissue plasminogen activator, tPA) was subsequently completed. The patient was transferred to the intensive care unit (ICU) after improved hemodynamics with a blood pressure of 105/68 mmHg on the norepinephrine infusion. Repeat FOCUS performed by an ultrasound fellowship-trained attending EP at two hours after systemic thrombolysis demonstrated continued evidence of RVD (Video 2), although a notable increase in TAPSE (Figure 8) and a decrease in peak TRV (Figure 9).

**Video 2:** Point-of-care echocardiography of massive pulmonary embolism performed two hours post-thrombolysis. Apical four-chamber view demonstrating right ventricular dilatation and McConnell’s sign.

**Figure 10:**
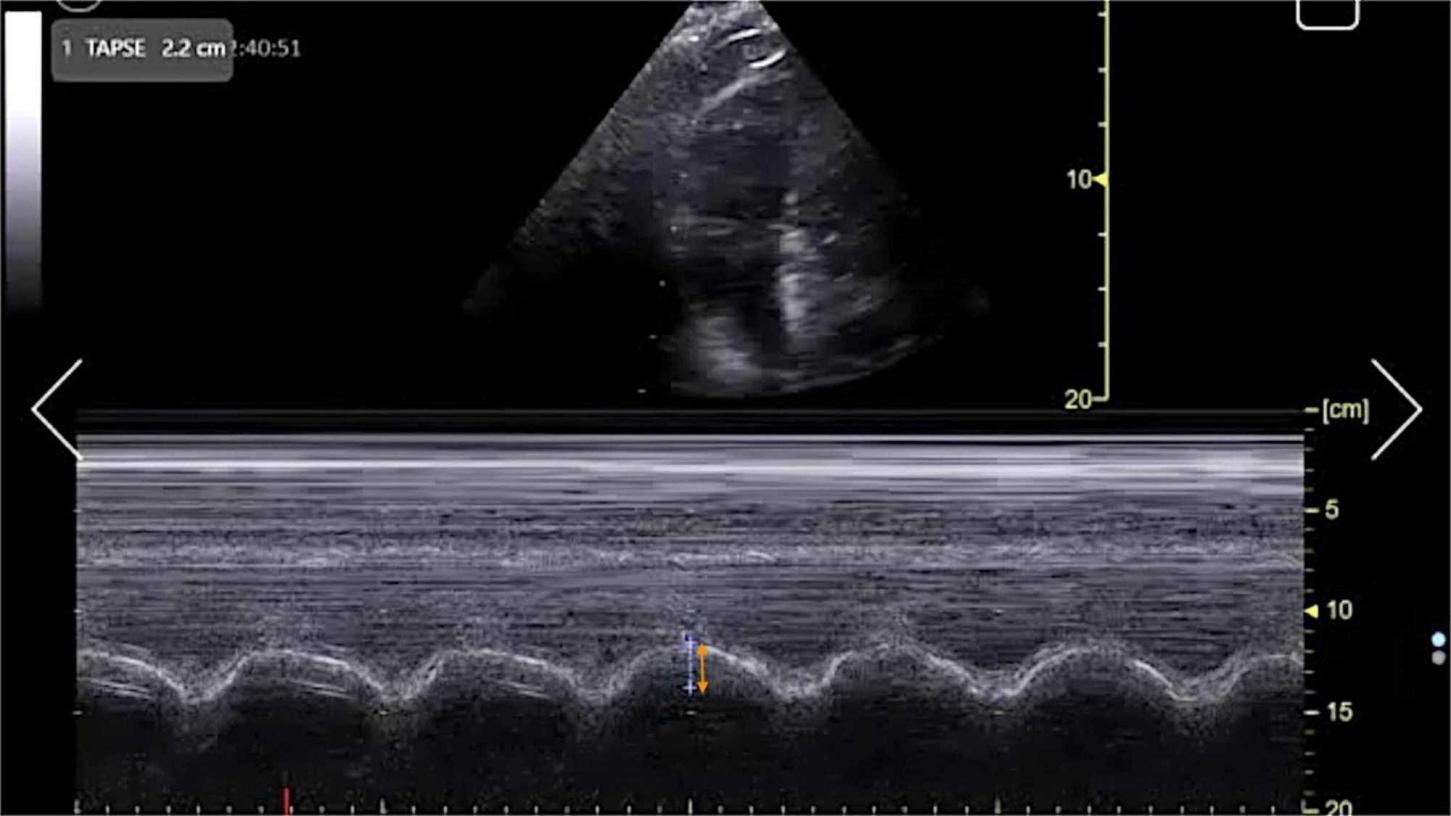
Point-of-care echocardiography 24 hours post-thrombolysis. Apical four-chamber view with M-mode tracing demonstrating TAPSE (orange arrow). TAPSE, tricuspid annular plane systolic excursion.

Radiology-performed bilateral lower extremity venous duplex ultrasound was obtained thereafter, which was significant for the presence of DVT in bilateral posterior tibial and peroneal veins. Twenty-four hours post-tPA intervention, the patient was on a heparin drip and weaned off vasopressors with a stable blood pressure of 110/58 mmHg, heart rate 83 beats per minute, respiratory rate 10 breaths per minute, and oxygen saturation of 100% on high flow nasal cannula at an FiO2 of 35% and flow rate of 40 L. An inferior vena cava filter was successfully placed by interventional radiology. Repeat FOCUS performed by an ultrasound fellowship-trained attending EP at 24 hours after systemic thrombolysis demonstrated a normalized TAPSE (Figure 10) and evidence of a substantial improvement in right heart function (Video 3). The FOCUS findings were communicated to the ICU team to assist in clinical decision making, and the patient was transferred to an ICU stepdown unit later that day. There were no major bleeding complications during his hospital course, but he did experience minor bleeding from the recent surgical incision site. The patient was discharged home on hospital day 5 on a novel oral anticoagulant regimen. 

**Video 3:** Point-of-care echocardiography of massive pulmonary embolism performed 24 hours post-thrombolysis. Apical four-chamber view demonstrating improvement of right ventricular strain.

## Discussion

The management of massive PE in the ED is of critical importance to lessening mortality burden. Point-of-care FOCUS is a critical tool in assisting with the timely diagnosis of PE [7-12]. The case described here again highlights the advantages of using FOCUS to identify signs of RVD in scenarios in which massive PE is clinically suspected, especially given that obtaining a definitive CTPA is challenging in the hemodynamically unstable patient. Our patient had RVD findings of right ventricular dilatation, interventricular septal flattening, and McConnell’s sign, similar to previously described cases [13,14]. Additionally, on initial presentation, the patient had an elevated peak TRV of 3.67 m/s and an estimated right ventricular systolic pressure (RVSP) of 58.9 mmHg (based on an estimated right atrial pressure of 5 mmHg on visualized inferior vena cava), which are both suggestive of RVD in the setting of acute PE [5,15]. At two hours post-thrombolysis, it is notable that there was a downward trending peak TRV of 2.57 m/s and an estimated RVSP of 31.4 mmHg, suggesting rapid improvement in RVD. Our patient had a pre-thrombolysis TAPSE of 12 mm which is significantly abnormal from established lower reference cutoff values, which vary from 16 to 20 mmHg [5,9,12,16]. The prompt identification of RVD by the EP is essential for risk stratification for acute PE [17].

TAPSE has been proposed as an ideal echocardiographic marker of RVD [5,16]. The longer-term mortality and prognostication significance of TAPSE in patients with acute PE have been described previously [18,19]. FOCUS was performed at three time intervals with TAPSE measurements as follows: TAPSE 12 mm immediately upon ED arrival, TAPSE 14 mm at two hours post-thrombolysis, and TAPSE 22 mm at 24 hours post-thrombolysis. To the best of our knowledge, this is the first case report to describe the dynamic resolution of TAPSE in EP-performed FOCUS for massive PE after receiving systemic thrombolysis. Notably, TAPSE and other echocardiographic findings of RVD had resolved within 24 hours after thrombolysis in correlation with the patient’s significant clinical improvement and hemodynamic stabilization. This case further emphasizes the importance of coordinated EP and intensivist collaboration in the management of acute massive PE.

## Conclusions

The high mortality burden of massive PE requires timely diagnosis and management by the EP. Serial bedside FOCUS can be instrumental in identifying signs of RVD, particularly TAPSE, for both diagnosis and management of acute massive PE. Further systematic research is warranted to investigate the short- and long-term benefits of EP-performed FOCUS in massive PE requiring thrombolysis.
